# Hyperplastic Polyps and Gastroduodenal Pseudomelanosis

**DOI:** 10.1155/2017/4270248

**Published:** 2017-11-15

**Authors:** Akanksha Agrawal, Deepanshu Jain, Vivian Arguello, Daniel Sher

**Affiliations:** ^1^Department of Internal Medicine, Albert Einstein Medical Center, Philadelphia, PA, USA; ^2^Department of Digestive Diseases and Transplantation, Albert Einstein Medical Center, Philadelphia, PA, USA; ^3^Department of Pathology and Hematology, Albert Einstein Medical Center, Philadelphia, PA, USA

## Abstract

Pseudomelanosis is a rare endoscopic finding of the upper gastrointestinal tract characterized by hemosiderin deposits in histiocytes of lamina propria. We report a case of 72-year-old lady on chronic oral iron supplementation diagnosed with gastric hyperplastic polyps with background pseudomelanosis of stomach and duodenum. Concomitant occurrence of gastric pseudomelanosis, duodenal pseudomelanosis, and gastric hyperplastic polyps has never been reported. Its presence in the absence of gastritis raises question if pseudomelanosis could be associated with hyperplastic polyp. With limited literature on its etiology and prognosis, these patients should be prospectively followed and reported to study the natural history of the disease.

## 1. Introduction

Pseudomelanosis is a rare endoscopic finding of the upper gastrointestinal tract characterized by accumulation of iron or hemosiderin in the histiocytes of lamina propria. This presumably benign pathology is associated with multiple clinical conditions like hypertension, chronic renal failure, diabetes mellitus, and long term intake of variety of medications like iron supplements and diuretics [1]. It is usually an incidental finding. Its concomitant occurrence with hyperplastic polyp has never been reported. The long term prognosis and sequela are not completely known. We present a case of a 72-year-old lady on chronic iron supplementation diagnosed as having pseudomelanosis of the duodenum and stomach and gastric hyperplastic polyps.

## 2. Case Report

**Figure 1:**
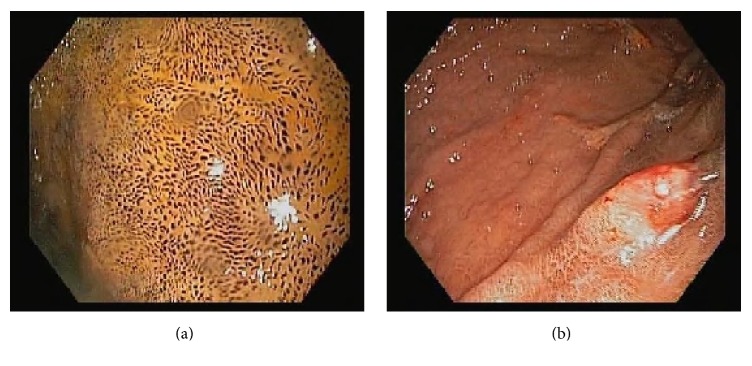
Endoscopic image showing brown-black speckled spots throughout the mucosa of the duodenum (a) and gastric polyp (b).

**Figure 2:**
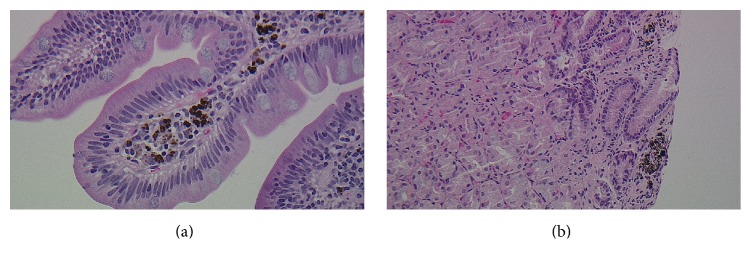
Hematoxylin and eosin stain (×400) of the gastric (a) and small intestinal mucosa (b) showing dark brown clumpy pigment present within histiocytes in lamina propria.

**Figure 3:**
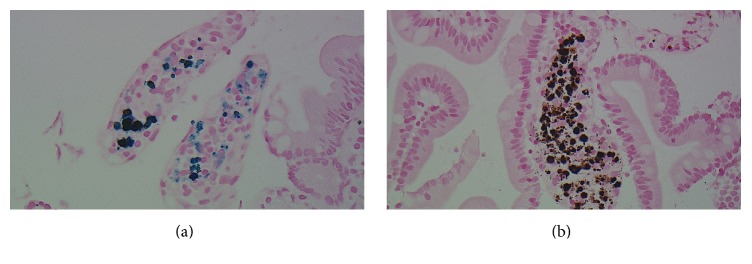
Panel of two images; Prussian blue stain (×600) with blue pigment deposition confirming the presence of iron within histiocytes (a). And Fontana Masson stain (×600) of the small intestine with brown-black pigment deposition consistent with melanin or melanin-like substance deposition in the macrophages (b).

A 72-year-old African American lady with history of diabetes mellitus, hypertension, chronic kidney disease, and iron deficiency anemia presented with an episode of coffee ground emesis. She had been receiving iron supplementation for about 4 years. Her vitals were stable and laboratory findings significant for anemia and kidney disease. She underwent esophagogastroduodenoscopy showing hyperpigmented mucosa with black speckled spots throughout the mucosa in stomach and duodenum (Figure 1(a)). Endoscopy also showed four polyps, three of them in gastric body, largest being 1.2 cm in size (Figure 1(b)). The mucosa overlying polyp was erythematous with old blood at the top of the polyps. The hematoxylin and eosin stain of the biopsy showed small intestinal and gastric oxyntic mucosa with dark brown clumpy pigment within histiocytes of the lamina propria (Figure 2). Prussian blue stain was positive for iron depicted as blue deposition in the histiocytes and Fontana Masson staining showed brown-black pigment deposition consistent with melanin or melanin-like substance (Figure 3). The biopsy of polyp was consistent with hyperplastic polyp with no evidence of gastritis. Immunohistochemical staining for* H. pylori* was negative. Given the endoscopic and pathological findings, she was diagnosed with gastric hyperplastic polyps in the background of pseudomelanosis of stomach and duodenum. She was advised to follow up for surveillance endoscopy.

## 3. Discussion

Pseudomelanosis was first described in duodenum by Bisordi and Kleinman in 1976 [2] and in stomach by Treeprasertsuk et al. in 2000 [3]. It is usually reported as an incidental finding in patients undergoing endoscopy for abdominal pain. It manifests endoscopically as peppery brown- black speckles on the mucosa. There are multiple conditions like chronic kidney disease, gastric hemorrhage, diabetes, and hypertension associated with pseudomelanosis. Also medication use like iron, diuretics, and hydralazine is correlated with PM, but no particular etiology has yet been elucidated. Melanocytes are not usually present in the GI tract. Pseudomelanosis occurs due to deposition of pigments like iron or hemosiderin in the histiocytes. A study by Giusto and Jakate tackled the unpredictable nature of iron stain in pseudomelanosis duodeni, where 18% patients were entirely positive and 64% partially positive for Perl's iron stain [4].

Pseudomelanosis has never been reported along with hyperplastic gastric polyps. Hyperplastic gastric polyps can be premalignant for gastric cancer [5], with dysplastic elements and focal cancers being found in 5% to 19% of hyperplastic polyps. Certain features like size greater than 1 cm and pedunculated morphology have been identified as risk factors for dysplasia in hyperplastic polyps [6]. Use of proton pump inhibitor (PPI) therapy is a known predisposing factor in the development of hyperplastic polyp. Our patient was using PPI chronically before the hospital admission.

Our patient's medical history, chronic iron supplements use, and endoscopic and histologic findings were consistent with pseudomelanosis of the stomach and the duodenum. Concomitant presence of hyperplastic gastric polyp in the absence of gastritis raises the concern for its association with pseudomelanosis, if any. Since this is a case report, no causality or association can be made between hyperplastic polyp and pseudomelanosis.

## 4. Conclusion

Pseudomelanosis of stomach and duodenum is a rare endoscopic finding, more common in older age group, and associated with certain clinical conditions and medication use but not reported before in the presence of gastric hyperplastic polyp. Such patients should be prospectively followed to determine the prognosis and the natural history of the disease.
